# Physiotherapists’ experiences of physiotherapy interventions in scientific physiotherapy publications focusing on interventions for children with cerebral palsy: a qualitative phenomenographic approach

**DOI:** 10.1186/1471-2431-12-90

**Published:** 2012-07-02

**Authors:** Ingalill Larsson, Michael Miller, Kerstin Liljedahl, Gunvor Gard

**Affiliations:** 1 Department of Health Sciences, Division of Physiotherapy, Lund University, Box 157, SE- 221 00, Lund, Sweden; 2 Habilitation & Assistive Technology Service, Region Skåne, Dockplatsen 26, SE -211 19, Malmö, Sweden

**Keywords:** Physiotherapy intervention, Cerebral palsy, Family-Centred Service, Motor learning, Phenomenography

## Abstract

**Background:**

Physiotherapy research concerning interventions for children with CP is often focused on collecting evidence of the superiority of particular therapeutic methods or treatment modalities. Articulating and documenting the use of theory, instrumentation and research design and the assumptions underlying physiotherapy research interventions are important. Physiotherapy interventions focusing on children with Cerebral Palsy should, according to the literature, be based on a functional and environmental perspective with task-specific functional activity, motor learning processes and Family-Centred Service i.e. to enhance motor ability and improve capacity so that the child can perform the tasks necessary to participate actively in everyday life. Thus, it is important to coordinate the norms and values of the physiotherapist with those of the family and child. The aim of this study was to describe how physiotherapists’ experiences physiotherapy interventions for children with CP in scientific physiotherapy publications written by physiotherapists.

**Methods:**

A qualitative phenomenographic approach was used. Twenty- one scientific articles, found in PubMed, strategically chosen according to year of publication (2001–2009), modality, journals and country, were investigated.

**Results:**

Three qualitatively different descriptive categories were identified: A: *Making it possible* a functional-based intervention based on the biopsychosocial health paradigm, and the role of the physiotherapist as collaborative, interacting with the child and family in goal setting, intervention planning and evaluation, B: *Making it work* an impairment-based intervention built on a mixed health paradigm (biomedical and biopsychosocial), and the role of the physiotherapist as a coach, leading the goal setting, intervention planning and evaluation and instructing family members to carry out physiotherapist directed orders, and; C: *Making it normal* an impairment-based intervention built on a biomedical health paradigm, and the role of the physiotherapist as an authoritative expert who determine goals, intervention planning and evaluation.

**Conclusions:**

Different paradigms of health and disability lead to different approaches to physiotherapy which influence the whole intervention process regarding strategies for the assessment and treatment, all of which influence Family-Centred Service and the child’s motor learning strategies. The results may deepen physiotherapists’ understanding of how different paradigms of health influence the way in which various physiotherapy approaches in research seek to solve the challenge of CP.

## Background

Cerebral palsy (CP) is the most common motor disability in children and covers a very heterogeneous group of disabilities with variations in severity, including ambulatory dysfunction, clumsiness and delayed acquisition of motor skills, often accompanied by disturbances in communication, cognition, perception and sensation [1]. The process of physiotherapy decision making in general includes assessment, goal-setting, planning and implementation of therapeutic, pedagogical and environmental interventions, followed by the evaluation of the results. The overall aim of interventions is to maximise function and minimise incapacity, and to modify the client’s environment to ensure their fullest possible participation in society thereby increasing autonomy and empowerment [2]. Physiotherapy research concerning interventions for children with CP during the past decade has focused on collecting evidence of the superiority of particular therapeutic methods or treatment modalities [3]. However no conclusive evidence has been found that one modality of treatment of children with CP is better than another in randomized controlled trials [3,4]. Essential aspects such as the expertise and skill of the physiotherapist, the interaction between the child and the therapist, the parents’ and the child’s satisfaction with the therapy, parental support, activities at home during leisure time and the child’s overall subjective well-being, have not been considered [3,5]. Articulating and documenting the use of theory, instrumentation and research design and the assumptions underlying the intervention are thus important and present a challenge to physiotherapists [3,6]. In this qualitative study, physiotherapists’ assumptions underlying research interventions are in focus. Some theoretical frameworks which focus on physiotherapy interventions for children with CP are illuminated below such as Family- Centred Service (FCS), the World Health Organization’s International Classification of Functioning, Disability and Health (ICF), and motor learning strategies.

Physiotherapy interventions in clinical practice and in research focusing on children with CP should not only be based on the perception of the child’s disability and the associated consequences from both a functional and environmental perspective, but also on the child’s own motivation, the family’s expectations, and the support they receive [3-11]. This approach is known as Family-Centred Service (FCS) and has been proven to be effective not only regarding the outcome for the child and his or her family as it influences the structure, process and outcomes of the physiotherapy intervention but also for the service delivery system [11-14]. FCS focuses on client-centredness [15] with the goal of enhancing and optimising capabilities, ensuring that the family and child have opportunities to participate in clinical decision making. Their desire and/or ability to participate may fluctuate due to socio-economic stressors and environmental and lifestyle factors. Thus instilling empowerment and creating power and control in the family and child is important [11-14,16]. From the FCS perspective, it is important that the child’s and their parents’ voices can be heard regarding goals, values and ideas connected to the intervention planning i.e. things they regard is important in their every-day life. Physiotherapeutic interventions must therefore coordinate the norms and values of scientists and clinically active physiotherapists with those of the client, in this case both the family and the child [17].

Different paradigms of health and disability lead to different strategies for the assessment and treatment of children with CP [6]. FCS requires a perspective in which disability is regarded as a social construction involving the interaction of the child with his or her environment [11-14]. ICF [18] is a conceptual biopsychosocial model of health. A child and youth version of this model, ICF-CY, has been developed [19]. Many physiotherapists working with children with CP incorporate the ICF-CY into practice as it focuses on health rather than the consequences of disease or disability. The components of body function and body structure, activity and participation interact dynamically with each other and with personal and environmental factors. The ICF-CY identifies relevant impairments, activity limitations and participation restrictions, and provides a conceptual framework for recognizing the effects of personal and environmental contextual factors on the components of health and can also be helpful in identifying primary goals and evaluating the effects of interventions [20-22].

Physiotherapy intervention strategies for children with CP vary, but the main aim is to enhance motor ability and improve capacity so that the child can perform the tasks necessary to participate actively in everyday life [3-10]. In physiotherapy, movement is understood as the fundament of an individual’s function, adapted to its purpose to achieve goals in relation to the surrounding environment [2]. Movement is the result of the interaction of the individual, the task and the environment. The movement patterns and the movement strategies used by individuals to achieve their goals are their own solutions to the motor problem in interaction with the environment [23]. Motor learning is based on the concept that learning is a process resulting in the capability to perform skilled actions that contributes to relatively permanent changes in behaviour. It cannot be measured directly, and is the result of experience or practice undertaken in order to learn new strategies for sensing and moving [24]. Learning is a process of acquiring knowledge about the world [25,26]. The socio-cultural context in which the action is assumed to be performed influences the child’s learning process and the child’s opportunity to develop strategies for action. The action requires interpretation and creativity, but is not always explicit or even conscious. A child acts in different situations depending on their knowledge, experience and understanding of the situation. Learning how to cope and how to be an active problem solver is based on previous experience and knowledge of the demands placed on the situation [27].

Task-specific functional activity and motor learning processes based on the child’s capability to learn should be emphasized in physiotherapy interventions for children with CP [27-31]. The environmental factors are important as these children may have difficulties in generalising movement strategies for different settings, i.e. the skill should be practiced in a real world setting [32]. To enhance motor learning and increase the child’s capability the physiotherapist shall firstly identify the environmental constraints and the child’s restrictions, modify the task if necessary, apply feedback, adjust the environment to promote performance, stimulate different strategies for problem solving, and offer new opportunities for the child to develop new solutions to a motor problem as an active problem solver [27-31]. Generally children learn cognitive and motor skills by training and through reasoning. Training implies acquiring habits of mind and behaviour that have been shaped by others, enabling the child to acquire the skills required to fit in. Regarding the child as being able to think and act according to his/her own capability is a better and a more empowering learning strategy than simple unreflective motor training. In this way the child develops autonomy and responsibility through reasoning [33].

The aim of this study was to identify and to describe how physiotherapists understand the theoretical assumptions of physiotherapy interventions from descriptions of interventions studies for children with CP, i.e. to identify variations in the underlying assumptions and theory of physiotherapy interventions focusing on CP.

## Methods

A qualitative phenomenographic approach was used. The aim of phenomenography is to identify and to describe various ways of experiencing and understanding a phenomenon. In phenomenographic research the term conception is synonymous with the term experience. Individuals are often focused and aware of the phenomenon they experience. However, they are not aware of the ways they experience it [26]. The phenomenon investigated in this study was physiotherapy interventions for children with CP, as described in previously published scientific articles, written by physiotherapists. Marton and Booth [26] states that an experience is a way of distinguish something from something and relate it to a context. The interest in phenomenography research is focused on the structural or referential aspects of the experience irrespective of whether it reflects the solution of a problem, the immediate conception, the action or, as in this study, the representation of physiotherapy interventions. Thus in a phenomenographic study data can consist of previously published scientific articles [25,26,34].

It is important to distinguish between the first- and second-order perspective in qualitative research [25,26]. If a researcher is interested in describing the essence of the phenomenon as an aggregated mental construction with the aim of interpreting the respondent’s statements and describing what the phenomenon *is*, the first-order perspective is used. In phenomenographic research, on the other hand, the objective is to elucidate the second-order perspective, i.e. describe the underlying causes of experiencing the world, the phenomenon and the situation. When writing scientific articles with the aim of testing or describing different physiotherapy interventions for children with CP, physiotherapists are aware of a number of aspects that are important to them. They discriminate their experiences and direct their awareness to these aspects of their intervention. Different combinations of these aspects contribute to the way in which physiotherapists experiences the concept of health and the world, influencing their experiences of what physiotherapy research for children with CP is about. This in turn leads to differences in *how* physiotherapy interventions for children with CP in various ways are experienced and understood [25,26].

### Material

PubMed was searched for articles with the keywords *physiotherapy* OR *physical therapy* AND *cerebral palsy* AND *treatment*. Limitations were patient age 0–18 years, human, clinical trials, English language, abstract available, published between 1 January 2001 and 31 December 2009. The inclusion criteria were: quantitative methods, currently accepted clinically based, traditional therapeutic motor interventions and physiotherapy. The exclusion criteria were: qualitative methods, reviews, lack of diagnosis of CP, and adjuncts to physiotherapy including surgical interventions, acupuncture, psychotherapy and pharmacotherapeutic interventions. Fifty-five articles were found to be relevant according to the search criteria.

At first the fifty-five articles were organised according to year of publication. Then the abstract of each article was read in order to find variations of the modalities used. The articles were the research was done were identified. A strategically selected sample of twenty-one articles was then chosen according to the maximum variation strategy [26]. This implies that each of the twenty-one articles [35-55] has variations in relationship to the others concerning year of publication, modality used, journals (with the aim of using well known relevant journals, commonly read by physiotherapists), and countries. The non-included articles were removed as they did not fulfil the maximum variation strategy and were not read in full.

· **Year of publication**: 2001–2009.

· **Modalities**: Functional therapy, strength training, strength training aided by electrical stimulation, Bobath therapy, hippotherapy, constraint-induced therapy, anti-spastic positioning, partial body-weight-supported treadmill training, functional electrical stimulation, electrical stimulation in addition to passive stretching, use of virtual reality, exercise training, intermittent versus continuous physiotherapy, balance training.

· **Journal**: Phys Ther, Clin Rehabil, Dev Med Child Neurol, J Altern Complement Medicine, Pediatrics International, Aust J Physiother, Pediatr Phys Ther, Arch Pediatr Adolesc Med, Pediatrics, Arch Phys Med Rehabil, Phys Occup Ther Pediatr.

· **Countries**: The Netherlands, Turkey, USA, Taiwan, Iran, UK, Sweden, Denmark, Australia, and Brazil [35-55].

### Analysis

The material was analysed in seven steps according to accepted phenomenographic procedure [56]. Dahlgren & Fallsberg [56] described the analysis process by using a metaphor:

Imagine that somebody is given an ordinary pack of playing cards and asked to sort them. Most probably the result would be four different groups of cards according to the four suits. A possibility is of course thirteen groups according to denomination. In phenomenographic research the task is to divide a number of dialogues (or written material, authors’ comment), but an important difference in comparison with the card sorting task, is the fact that the researcher does not previously know the categories according to which the task could be solved. The result instead consists of finding and defining the existing meaning in the dialogues (or written material, authors’ comment) according to which they can be grouped [56 p. 152].

The first author analysed the articles in continuous discussion with the other authors within every step. Questions (see below) were formulated for every step of the analysis and directed to the material in order to sharpen the analysis.

1. **Familiarisation** - The articles were carefully read several times, in order to become acquainted with the material and to obtain knowledge in relation to the focus and strategy of the intervention.Questions: How is the primary focus and strategy in the intervention described? Is the disability described as resident within the individual and the intervention focused on correcting the child’s weaknesses or problems i.e. impairment-based strategy? Is the disability described as due to complex circumstances within the environmental context and the intervention focused on functional goals and practice of motor abilities within a meaningful environment i.e. functional-based strategy?

2. **Condensation** - The quotations that described the physiotherapeutic intervention were selected and marked with a highlighter pen; these were then cut out of the material with a pair of scissors. The quotations were marked with the article to which they belonged. At this time the material consisted of parts of the whole.Question: How are the interventions described in the background, methods, results and discussion sections?

3. **Comparison** - These quotations were compared to identify sources of variation and agreement between the ways the interventions were described.Questions: Which aspects of the intervention are described and which are not? How does this aspect relate to the intervention used? Are there different ways to use some aspects?

4. **Grouping** - Quotations that were similar in their way of experiencing the interventions were grouped for preliminary classification. Three different groups were identified and no quotations were omitted. The meaning of a quotation lies in the utterance itself, but it should also be understood in relation to its original context. Thus, the articles from which the quotations were taken were read once again. Each quotation was interpreted in two contexts: in relation to the article from which it was taken, and in relation to the group of experience to which it belonged.Questions: Do the quotations use the same aspects in the same way? Do the quotations exclude some aspects and incorporate others? What are the differences between these quotations? Has this quotation another aspect not previously discovered? How is the intervention experienced in these quotations? Are there any critical variations in how the physiotherapy intervention is experienced?

5. **Articulation** - The essence of the similarities in each qualitatively different and non-overlapping descriptive category was described by a limited and central content reassuring the critical variations in the experiences. The quotations from one article together with quotations from others, form a description of the different ways in which physiotherapy interventions are experienced.Questions: What content do the quotations that belong to this descriptive category describe? Can this quotation belong to more than one descriptive category?Steps 3–5 were repeated several times. The authors discussed relevance, similarities and differences before the analysis was deemed to be satisfactory.

6. **Labelling** - Each descriptive category was assigned a tailored linguistic expression i.e. the headings of the descriptive categories.Question: Which expression can be used to make this descriptive category understandable in relation to how the physiotherapy intervention is experienced?

7. **Contrasting** - The final descriptive categories were compared with regard to similarities and differences i.e. critical variations. The descriptive categories represent a range of more or less complex experiences of physiotherapy interventions.In phenomenography the experience of the phenomenon forms a whole through separation, differentiation, demarcation and organisation of the material, which requires analysis and some interpretation of the material [26,56]. The seven steps procedure implies that each article was analysed resulting in iterative evaluation back and forth between the whole and the different parts of each article. The experience described in one article cannot be understood in isolation from the others as the descriptive categories describes a collective and not an individually level of experiences.

## Results

**Figure 1:**
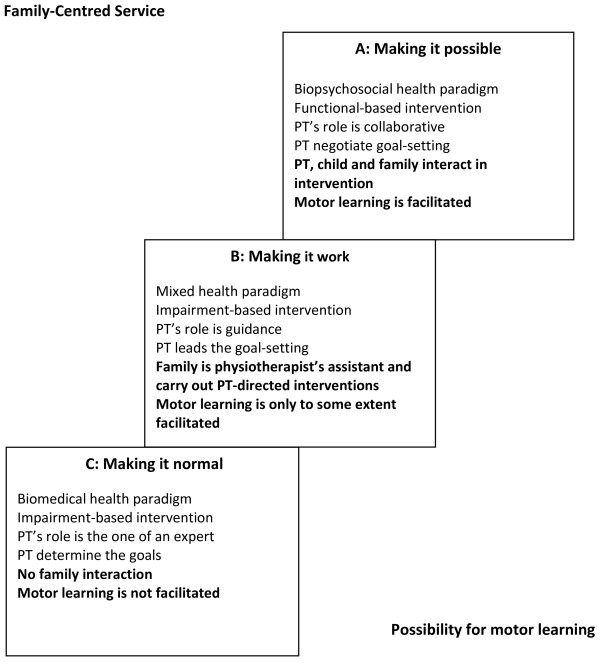
**The outcome space.** Illustration of the outcome space of physiotherapists’ experiences of physiotherapy interventions for children with CP in a strategically selected sample of 21 scientific published articles (2001–2009), written by physiotherapists, with implications for family-centred service and possibilities for motor learning. PT = physiotherapist

Three qualitatively different descriptive categories were identified in twenty-one scientific articles [35-55] regarding physiotherapists’ experiences of physiotherapy interventions for children with cerebral palsy in scientific, published articles, written by physiotherapists. The descriptive categories have a hierarchical relation with each other which is illustrated in the outcome space (see Figure 1). According to Marton & Booth [26] there can be a standard, a specific way of experiencing the phenomenon, that can be more preferable than others i.e. is linked to theory as elucidated in the background section in this study, and more complex and comprehensive in relation to the way the phenomenon is experienced. In this study, descriptive category A is more preferable, complex and comprehensive than descriptive category B, which in turn is more preferable, complex and comprehensive than descriptive category C (see Figure 1).

### A: Making it possible

The experience of physiotherapy interventions in scientific articles is based on the biopsychosocial paradigm of health from the perspective of empowerment. The intervention is functionally based and is directed towards reducing the child’s limitations in activity and restrictions on participation in the context in which the child functions on a daily basis. The physiotherapist uses the child’s environment, the parents’ participation, and the child’s creativity and resources to solve the motor problem, thereby empowering the child and parents to become active participants in the intervention process. Goals are discussed and set according to the needs of the family and child.

The collaborative goal-setting process involved the child’s physiotherapist discussing the problem with the child (if appropriate), the parents, carers, teachers, or nursery nurses, setting goals with them including establishing their base-line measurements, undertaking the intervention, and after a set period (three months) evaluating the goals to ascertain to what extent they have been achieved [36, p. 7]

The short-time goals are practiced in various natural settings..//.. practice takes place in natural situations (mostly at home or outdoors ..//..) The therapist and parents discuss how, when, and where to practice. They also discuss the amount of assistance, the reduction of assistance, the time of day that is most practical for practising the specific skill (fit into the daily routines..//..). The parents, child, and therapist together evaluate the goals [35, p. 1539].

### B: Making it work

The experience of physiotherapy interventions in scientific articles is influenced by a mixed health paradigm in which the physiotherapist tries to balance the impairment-focused interventions based on the biomedical paradigm and functional-focused intervention founded on the biopsychosocial paradigm. The impairment is experienced as being the key factor in the child’s disability and the main focus of the intervention is on correcting the child’s disability influenced by the biomedical paradigm. However, the goals are focused on both increasing body functions and on increasing the child’s activity and participation and the goals are thus also influenced by the biopsychosocial paradigm of health. The intervention takes place in the child’s environment and with parental support. In the intervention the child practises the exercises defined and controlled by the physiotherapist. The parents assist the physiotherapist who instructs, supervises, controls and evaluates the intervention and trains the child and their parents in how to carry out the exercises.

Each training session consisted of a 3 to 5 minutes aerobic warm-up, followed by plantarflexor stretches..//.. The plantarflexor strengthening exercises were then performed..//. The functional scores increased slightly through the period of the study although the differences failed to reach significance. [55, p. 430 and 433]

A home-exercise program, consisting of functional and play activities, was to be performed while wearing the mitt. His mother was instructed to encourage his use of the mitt at home and during appropriate activities. [38, p.1462]

At the end of each day, each child went home with an exercise program to practice with the involved upper extremity (without any restraint for 1 hour during the evening…//… and parents completed activity logs to monitor compliance. [46, p.365]

### C: Making it normal

The experience of physiotherapy interventions in scientific articles is influenced by the biomedical health paradigm with normality as a standard which, with a dualistic perspective considers parts of the body as an object of scientific study. The physiotherapist uses his or her professional expertise to plan the intervention, which is strictly impairment-based and focused on the impairment and its effect on the child’s body function. The child is exposed to the intervention which is based on repetition and facilitated by devices or physiotherapist’s hands-on treatment. The intervention takes place in a clinical environment with goals mostly related to the body function component of the ICF-CY. Few goals are related to the activity component and none are related to the component of participation. Neither the family nor the child participates in goal-setting or evaluation.

Patients were supported by a physiotherapist at a straight sitting position as hips were abducted at nearly a 45° angle and externally rotated, and the knees were extended to 90° of the ankles. Patients were kept in this position for 20 min without changing the degree of support. The head was held in a neutral position in order to prevent asymmetrical tonic neck reflex. [44, p.442]

A licensed physical therapist and two research assistants facilitated the children’s gait pattern on the treadmill during the training process. The treatment goal was to reproduce a normal gait cycle throughout the sessions: attention was paid to appropriate gait kinematics, emphasizing heel strike at initial contact, knee extension at stance phase, and hip extension at terminal stance. One training facilitator was positioned behind the child to provide stabilization at the hip while the other facilitators assisted leg movements as needed in order to assist the child to achieve normal gait kinematics [51, p. 6].

## Discussion

From scientific physiotherapy publications three qualitatively different descriptive categories were identified that described various ways in which physiotherapists’ experiences physiotherapy interventions for children with CP. In phenomenography individuals are considered as carriers of fragments of different ways of experiencing the phenomenon. This implies that the descriptive categories are a theoretical description of the variations of experiences at a collective, not individual, level [25,26]. Critical variations in the way in which physiotherapy interventions were experienced were identified in the descriptive categories regarding health paradigm, intervention focus, the role of the physiotherapist and the goal-setting procedure, all of which have implications for FCS [12-14] and strategies for motor learning [27-32] (see Figure 1).

## Results

The descriptive category A: *Making it possible*, is based on the biopsychosocial paradigm and takes all the interacting components in the ICF-CY [19] into consideration. In this descriptive category the standpoint is that the environment has to be modified in order to make it possible for the child and their families to participate in activities that are important to them. A counter point position would be that it is the child that must be adjusted, i.e. improved muscle strength or increased joint range of motion in order to manage the environmental demands. In descriptive category A the physiotherapist adopts a collaborative and empowering role which has been supported in previous studies concerning physiotherapy interventions [57-59]. This research approach, which is centred on the child and family, is based on a holistic view of the child and family with the conception of physiotherapy knowledge as the ability to interact and use professional competence in the intervention process [59]. Jensen et al. recognized this interactive approach as a keystone in expert physiotherapy [60]. The role of the parents is to be active partners and to help the child to incorporate new treatment-related behavior into everyday life [14,16]. The biopsychosocial paradigm facilitates goal-setting with the focus on the child’s potential in every-day life, which are relevant according to the components activity and participation in the ICF-CY model. In this client-centred goal-setting [61] the power and control over interventions and evaluation are shared between the child, the family and the physiotherapist, thereby employing the paradigm of FCS [12-14], empowerment [16], and strategies for motor learning [27-31]. This is important for both the child and the family and will make therapy in clinical settings and in research more effective and meaningful [61-65]. The child’s motor learning process is encouraged by stimulating the child to be an active problem solver during the physiotherapy intervention, thereby enabling the child to learn motor strategies to achieve a higher degree of independence in their environment [27-32]. According to Valvano the child’s practice of meaningful tasks based on the child’s individual ability in a true environmental situation is essential for the child’s development of motor control and coordination [28].

In descriptive category B: *Making it work*, a mixed paradigm of health was identified. This is due to the influences from both the biomedical paradigm, which focuses on the impairment and is directed towards correcting the child’s disability, and the biopsychosocial paradigm, as the intervention is also directed towards making a difference in the child’s ability to participate actively in a particular environment. Valvano describes this as “an impairment-focused intervention with activity-focused goals” which has been proposed as a common combination in physiotherapy interventions for children with CP [28]. The role of the physiotherapist derived from the descriptions interpretation of the results encompassed in this descriptive category is to train the child and family, and to give special instructions as to what they should do, i.e. the role of a coach. Research has shown that the parents and the child have little influence over the intervention and its goals when physiotherapists adopt this mixed paradigm because the goal-setting procedure and the intervention are solely physiotherapist led [57,61-65]. This is also in accordance with the experiences of parents and children with CP in decision making in community-based paediatric physiotherapy [66] and with a study describing physiotherapists’ experiences of client participation in physiotherapy interventions as *guidance*[57]. In adopting a mixed biomedical/biopsychosocial paradigm the physiotherapist takes full responsibility for enhancing health, avoiding risks and reducing complications without fully inviting the child and families to be active in the process. This can be recognized as an ability to follow the demands on the profession [59]. The demands are many and sometimes divergent. The health-care system demands that physiotherapists provide good clinical services according to evidence and/or well-tried clinical practice. Moreover there may be demands for health- care cost control and cost reduction, while at the same time demands to respond to the needs and expectations of the child and family [17]. In research these different demands may be contradicted. In descriptive category B, a major input as to how the child tackles motor problems comes from instructions given by the physiotherapist. Physical guidance can be used early in practice in order to help the child to feel the movement and to facilitate the development of the child’s action plan [28]. However, the criticism is that optimal motor learning will not be achieved unless the child is actively engaged in solving the problem to meet the challenge of the task [27-32].

Descriptive category C: *Making it normal*, is embedded in a biomedical paradigm as the research intervention is directed towards the child’s disability with the innate understanding that the environment, the child’s own motivation and the family’s needs, are not adequately considered. The descriptive category C differs considerably from the descriptive categories A and B. The focus of the intervention on only specific impairment aspects implies a deductive approach [57,59]. Different motor impairments within the spectrum of CP can be treated with different therapeutic exercises, but the transferred functional benefits of impairment-focused interventions have yet to be proven effective. Thus, strictly impairment-focused physiotherapy research interventions for children with CP may not be beneficial for the child to optimally function in daily life. According to Anttila et al. [3] and Damiano [4] physiotherapists should consider the functional task and not simply treat the impairment. In the biomedical paradigm the role of the physiotherapist is that of an expert who has full control and power over the intervention process. This authoritarian approach, centred on the physiotherapist as an authoritative expert, has been described previously as one approach in physiotherapy interventions in clinical practice [57,58]. The physiotherapist is regarded as being the best suited to understand the full consequences of the disability and to determine the needs of others from their own perspective as opposed to those of their clients [57,58]. The goal-setting procedure is centred on the physiotherapist [61] and the child and parents are marginalized and not invited to take part in the process at all. Instead of being active participants in the process the child and family are reduced to powerless recipients of medical decisions and treatment [16]. Children’s self-confidence and self-esteem can depend on how well they understand the reason behind physiotherapy interventions, in other words, why they have to do the exercises [67]. This is important as children’s understanding also affects their ability to use their capacity in the challenges they face in everyday life, and thus their motor learning processes. The use of biomedical impairment-focused interventions in clinical settings, the lack of FCS, and the physiotherapist’s failure to enable the child to try to change his or her movement strategy contradicts the process of motor learning [27-32].

### Further considerations

We may assume a priori that studying relevant articles, commonly read by physiotherapists describes the underlying view of disability and health that physiotherapists use. This influences assumptions when conducting research within the area of CP, and thus different experiences of physiotherapy interventions in research can be described. The family puts their trust in the professional competence of the physiotherapist and therefore should be assured of getting the best treatment available for their child. Thus, it is vital that the physiotherapy profession continues to study physiotherapy interventions for these children with appropriately designed clinical trials [17]. However physiotherapists are affected by their own experiences which in turn are imbedded in paradigms that affect the physiotherapists choice of research strategy, treatment and evaluation [25,26]. The biomedical dualistic paradigm of disability and health is commonly adopted in physiotherapy research. It is useful in models of the human body, as it enables the researchers to study separate aspects of disability, and contributes to the description of its function in quantifiable terms. Thus physiotherapists’ experiences of physiotherapy interventions in research as found in this study, may be entrenched in the routines and ready-made solutions that permeate the tradition which is taken for granted [68].

Theory and assumptions about humans, health, the world, knowledge, science and physiotherapy together with the physiotherapists’ personal perspectives on health and disability are related to the practical and theoretical knowledge they use in their interventions in clinical practice and research [69,70]. Higgs et al. [71] state that knowledge is dynamic and that theoretical and practical perspectives coexist, are interconnected and interdependent, and that they are intertwined in interventions. The physiotherapy profession has expertise in body function and structure from a functional point of view. Physiotherapists also have profound knowledge on how the disabilities associated with CP, i.e. problems in sensory-motor development and the development of cognitive functions, contribute to the child’s ability to perform the task in the environment, and they should therefore base their research interventions on this knowledge [28]. Rosenbaum [14] argues that it is time to consider the developmental aspects of the child, as well as the family dimensions. This requires that physiotherapists not only should have focus on the child’s impairment in itself but also take more consideration of the child and family’s daily challenges and well-being. In research this is a challenge. However, the objective of interventions is to increase the child’s competence in their social and physical environment, and this requires the active participation of both the child and the parents [3-14,27-32]. The family may sometimes experience the need for, in their eyes, a physiotherapy expert who will tell them explicitly what to do. This may be grounded in their previous experiences of physiotherapy as a profession that provides authoritative instruction which may result in a transfer of decision making to the physiotherapist. Thus in these cases it may be difficult to establish a true collaborative relationship both in research and in clinical practice. Such parents may need a highly competent physiotherapist who can facilitate cooperative decision making [72]. Physiotherapists should be able to satisfy the parents’ needs for support and information using empowerment and collaboration and should not take complete control. Parents know what is best, and want the best, for their child based on their own unique view of life and parenthood, but they often require support to cope with their child’s disability and its impact on everyday life. The parents should be instructed and supervised, but they must also be encouraged as competent parents. Physiotherapists need to identify ways to help these children and their families to cope in daily life. Our standpoint is that physiotherapists should recognize that it is a professional skill to listen and learn from their clients. Furthermore we believe that the overall goal of physiotherapy interventions for children with CP is to encourage the involvement of the family and to create challenging strategies to enable the child to maximize his/her potential and autonomy in society, including the ability to solve problems and take control over their lives. Physiotherapists have to know how to create empowerment in the child and their family and together with them create new ways to control and handle limitations. The field of physiotherapy embraces knowledge within the areas of pedagogics and didactics and therefore more research concerning the pedagogical aspects of physiotherapy interventions for children with CP, is required.

### Methodological aspects and limitations

In health care it is essential to recognise how phenomena can be experienced and understood in qualitatively different ways by health-care professionals [56-58]. Thus, phenomenography can be an essential tool for the exploration of theoretical foundations and conceptual parameters of the discipline of physiotherapy [57,58]. The phenomenographic approach is an interesting though to date seldom used way of analysing previously published material. The articles analysed in this study described different therapeutic methods and treatment modalities in physiotherapy interventions in research, thus variations were expected in the way in which physiotherapy interventions were experienced. A phenomenographic analysis was used as a powerful tool to identify these variations. Once again, we want to point out that this study identifies the variations of *collected experiences* as they appear in the articles studied [25,26] which may not be wholly representative of how the individual authors actually experience physiotherapy intervention in their everyday clinical practice. A critical assessment of physiotherapy interventions in clinical practice is beyond the scope of this article.

The category system of a phenomenographic study is not definitive, as the results are derived from a limited number of sources, although the variation of experiences within the investigated material can be described. Other descriptive categories, not found in this study, may have been revealed in a larger sample and other material. However, the phenomenographic approach assumes some degree of transferability and that the same descriptive categories can be found in other similar sources [26].

The trustworthiness of the results in a qualitative study is dependent on the researchers’ methodological skill and competence. The researchers’ understanding and awareness of the phenomenon being investigated and of the context in which the phenomenon can be experienced and understood is important [26,73]. However, this can also be a limitation of this study. In the background section references from acknowledged researchers within the field of CP interventions and/or motor learning elucidate our understanding of the phenomenon. The first author in this present study has considerable clinical experience in treating children with CP and has shared the physiotherapeutic framework with the authors in the analysed articles. The results can be influenced by this pre-understanding, as well as our understanding of reality from our own cultural and ideological understanding of the phenomenon [73]. The human factor is both the great strength and the fundamental weakness of qualitative research and analysis. Following the phenomenographic approach, we are convinced that when the first author read the articles she probably experienced them according to how she herself experiences the physiotherapy interventions for children with CP. We were aware of this throughout the analysis and discussed it on several occasions during the analysis process.

Triangulation is often used in qualitative research to indicate that more than two methods or sources are used in a study with a view to double- or triple-checking results. Data triangulation, i.e. the use of a variety of data sources in studying the same phenomenon, using the same method is one way of strengthening the results and increasing trustworthiness in a qualitative study [73]. In a previously published study [57] we conducted a phenomenographic analysis of physiotherapists’ experiences of client participation in physiotherapy interventions. Even if there are points of similarities between the results in these studies the perspectives in the studies were not the same and the context was different. In phenomenography the experience is always related to the context [26] and thus triangulation has not been done.

The trustworthiness and reasonableness of a phenomenographic study are related to how well the results correspond to reality and how logical and understandable the results are [26]. The collection of the data used in this study is documented by a detailed description of how the articles were selected in order to obtain a broad sample according to the maximum variation strategy. To ensure trustworthiness quotations were used in illustrating each descriptive category [73] and the articles to which they belonged are presented in the reference list [35-55]. A pragmatic criterion can be used to address practical concerns about truth in qualitative studies [26,73]. The practical consequences of this study may be that physiotherapists conducting research within the area of children with CP recognize the different experiences of physiotherapy interventions described in this study. They can reflect over the health paradigm they use and how this may influence their research and what this may imply.

## Conclusions

Using a phenomenographic approach three qualitatively different descriptive categories of experiences of physiotherapy interventions for children with CP, described in scientific physiotherapy articles written by physiotherapists, have been identified. The descriptive categories identified were: A: *Making it possible*, B: *Making it work*, and C: *Making it normal.* Critical variations were found between the three descriptive categories according to health paradigms, intervention focus, goal-setting, the role of the physiotherapist, FCS and motor learning strategies. The results may deepen physiotherapists’ understanding of how different paradigms of health influence the way in which various physiotherapy approaches in research seek to solve the challenge of CP and may facilitate the design of future quantitative and qualitative studies on physiotherapy interventions.

## Competing interests

We declare that we have no competing interests.

## Authors’ contributions

IL conceived and designed the study with input from KL and GG. IL also performed the search, collection and the strategic selection of the papers used in the study. The initial analysis was done by IL in continuous discussion with KL, GG and MM. IL and MM have structured, aligned and drafted the manuscript. All authors read and approved the final manuscript.

## Pre-publication history

The pre-publication history for this paper can be accessed here:

http://www.biomedcentral.com/1471-2431/12/90/prepub
